# Conditional survival to assess prognosis in patients with chronic lymphocytic leukemia

**DOI:** 10.1007/s00277-024-05627-w

**Published:** 2024-02-03

**Authors:** Pascal Schlosser, Annett Schiwitza, Jonas Klaus, Stefanie Hieke-Schulz, Katarzyna Szarc vel Szic, Justus Duyster, Martin Trepel, Katja Zirlik, Martin Schumacher, Rainer Claus

**Affiliations:** 1 Institute of Genetic Epidemiology, Faculty of Medicine and Medical Center, University of Freiburg, Freiburg, Germany; 2 Department of Epidemiology, Johns Hopkins Bloomberg School of Public Health, Baltimore, MD USA; 3 Centre for Integrative Biological Signalling Studies (CIBSS), University of Freiburg, Freiburg, Germany; 4 Hematology/Oncology, Faculty of Medicine, University of Augsburg, Augsburg, Germany; 5 Department of Hematology, Oncology and Stem Cell Transplantation, University Medical Center Freiburg, Freiburg, Germany; 6 Institute of Medical Biometry and Medical Informatics, Faculty of Medicine and Medical Center, University of Freiburg, Freiburg, Germany; 7 Roche Pharma AG, Grenzach-Wyhlen, Germany; 8 Tumor- Und BrustZentrum Ostschweiz, Chur, Switzerland; 9 Pathology, Faculty of Medicine, University of Augsburg, Augsburg, Germany; 10 Faculty of Medicine, Comprehensive Cancer Center, University of Augsburg, Stenglinstr. 2, 86156 Augsburg, Germany

**Keywords:** CLL, Conditional survival, Prognostic biomarker, Mortality, *IGHV* mutation status

## Abstract

Biomarkers in chronic lymphocytic leukemia (CLL) allow assessment of prognosis. However, the validity of current prognostic biomarkers based on a single assessment point remains unclear for patients who have survived one or more years. Conditional survival (CS) studies that address how prognosis may change over time, especially in prognostic subgroups, are still rare. We performed CS analyses to estimate 5-year survival in 1-year increments, stratified by baseline disease characteristics and known risk factors in two community-based cohorts of CLL patients (Freiburg University Hospital (*n* = 316) and Augsburg University Hospital (*n* = 564)) diagnosed between 1984 and 2021. We demonstrate that 5-year CS probability is stable (app. 75%) for the entire CLL patient cohort over 10 years. While age, sex, and stage have no significant impact on CS, patients with high-risk disease features such as non-mutated *IGHV*, deletion 17p, and high-risk CLL-IPI have a significantly worse prognosis at diagnosis, and 5-year CS steadily decreases with each additional year survived. Our results confirm that CLL patients have a stable survival probability with excess mortality and that the prognosis of high-risk CLL patients declines over time. We infer that CS-based prognostic information is relevant for disease management and counseling of CLL patients.

**Supplementary Information:**

The online version contains supplementary material available at 10.1007/s00277-024-05627-w.

## Introduction

Chronic lymphocytic leukemia (CLL) is a clinically heterogeneous hematologic malignancy with variable outcomes [1, 2]. While some patients may have a prolonged survival without needing treatment, others experience a rapidly fatal disease course despite receiving highly effective therapies [3–5]. Improving patient management and treatment strategies requires reliable biomarkers to predict prognosis, disease progression, and treatment responses.

Advancements in understanding the molecular mechanisms and detailed clinical characterization have led to the identification of numerous prognostic and predictive biomarkers that complement the classic clinical staging classifications [6, 7]. Cytogenetic and molecular genetic aberrations like deletions in chromosomes 11q and 17p, *TP53* mutations, and the immunoglobulin heavy chain variable gene (*IGHV*) mutation status play a crucial role in prognosis estimation and treatment respons [8, 9]. Composite prognostic scores like the CLL International Prognostic Index (CLL-IPI) integrate genetic, biochemical, and clinical parameters to predict survival differences. However, these models have limitations as they fail to consider how prognosis may change over time [10–13]. From a patient’s perspective, the probability of surviving another *t* years when she/he has already survived *s* years might be more relevant than a static prediction. Conditional survival (CS) analysis, which takes into account how long an individual has already survived, offers dynamic prognostic information about changes in survival probability over time. For many cancers, CS is reported to increase over time [14]. In contrast, CLL has shown remarkable stable survival estimates even over a 10-year period following diagnosis. However, it is unclear whether this stability applies to all clinical stages or risk groups [15].

To address these knowledge gaps, we performed CS analyses in CLL patients with different risk profiles to improve prognosis estimation in patient subgroups by accounting for years already survived [16]. As treatment options evolve, incorporating dynamic prognostic information becomes increasingly crucial for guiding clinical practice and improving patient outcomes in CLL.

## Material and methods

### Patient cohorts and data collection

Data from CLL patients were collected independently from two German university hospitals, including baseline characteristics, clinical parameters, and biological and molecular markers. Three hundred and sixteen CLL patients were included at the Department of Hematology, Oncology and Stem Cell Transplantation at the University Freiburg Medical Center between December 1984 and April 2014, and 564 CLL patients were included at the Augsburg University Hospital between January 1999 and January 2021. The diagnosis of CLL and response to therapy were assessed according to the International Workshop on Chronic Lymphocytic Leukemia (iwCLL) guidelines [17]. All diagnostic variables were determined at the time of diagnosis or first presentation. Data on demographics, clinical and molecular biological parameters, and disease progression were collected from local clinical information systems, digitized reports from external practices and clinics, and the cancer registries of the Comprehensive Cancer Center Freiburg (CCCF) and Augsburg University Hospital.

Data collection was approved by the local ethics committee, and written informed consent was obtained from all patients from the University Freiburg Medical Center. For patients of the Augsburg University Hospital, anonymized retrospective analysis of data is permitted without informed consent according to the Bavarian Hospital Act (BayKrG) in the version of March 28, 2007 (GVBl. S. 288, BayRS 2126–8-G Art. 27 Abs4). The study including data collection and analyses was performed according to the terms of the Declaration of Helsinki.

### Assessment of biological and molecular biomarkers

Biological and molecular biomarkers were assessed according to local diagnostic standards (described in the Supplement).

### Survival analysis

Overall survival (OS) was calculated from the date of initial diagnosis until the date of death from any cause. When no event of interest occurred, observations were censored at the time the patient was last seen alive or at the latest on July 1, 2017, and March 1, 2021, for Freiburg and Augsburg, respectively. OS rates were estimated using the Kaplan–Meier method [18]. Conditional survival (CS) estimates stratified by covariables were based on Cox proportional-hazards model in the corresponding landmarked dataset [16]. OS rates were compared using the log-rank test. CS is defined as the probability of surviving additional time *t* after the patient has already survived a certain time *s*:

$$\text{CS}(t\vert s)\hspace{0.17em}=\hspace{0.17em}\frac{S(s+t)}{S(s)}.$$

CS was calculated using landmarks s = 0 to s = 10 years.

## Results

### Baseline patient characteristics

Data collection was performed for a total of *n* = 880 CLL patients of two community-based cohorts (Table 1; Freiburg University Medical Center: *n* = 316 CLL; Augsburg University Hospital: *n* = 564).

**Table 1:** Data collection for a total of *n* = 880 CLL patients of two community-based cohorts

		Freiburg Medical Center		Augsburg Medical Center		Combined cohort	
Number of patients		316		564		880	
Age at initial diagnosis	Median in y (iqr)	62 (53–69)		69 (61, 76)		67 (58, 74)	
	Age ≤ 65 at ID	204	65%	208	37%	412	47%
	Age > 65 at ID	112	35%	356	63%	468	53%
Sex	Male	209	66%	361	64%	570	65%
	Female	107	34%	203	36%	310	35%
Follow-up	Mean in y	10.2		5.7		7.3	
	Median in y (iqr)	8.7 (5.5–14)		4.4 (1.8, 8.4)		6.0 (2.7, 10.6)	
Rai stage	0	116	37%	198	35%	314	36%
	I–IV	197	62%	252	45%	449	51%
	Not available	3	1%	114	20%	117	13%
Binet stage	A	136	43%	358	63%	494	56%
	B/C	85	27%	204	36%	289	33%
	Not available	95	30%	2	1%	97	11%
*IGHV* mutational status	Mutated	175	55%	33	6%	208	24%
	Unmutated	114	36%	56	10%	170	19%
	Not available	27	9%	475	84%	502	57%
Serum β2-microglobulin	≤ 3.5 mg/dL	147	47%	0	0%	147	17%
	> 3.5 mg/dL	129	41%	0	0%	129	15%
	Not available	40	13%	564	100%	604	69%
del(17p)	Positive	21	7%	11	1%	32	4%
	Negative	281	89%	44	5%	325	37%
	Unknown	14	4%	509	90%	523	59%
*ZAP70* CpG + 223 methylation	< 20%	138	44%	0	0%	138	16%
	≥ 20%	127	40%	0	0%	127	14%
	Not available	51	16%	564	100%	615	70%
CLL-IPI	Low risk (0)	84	27%	0	0%	84	10%
	Intermediate risk (1)	87	27%	0	0%	87	10%
	High risk (2)	79	25%	0	0%	79	9%
	Very high risk (3)	15	5%	0	0%	15	2%
	Not available	51	16%	564	100%	615	70%
Treatment	Treatment	212	67%	272	48%	484	55%
	No treatment	104	33%	292	52%	396	45%
CIRS	≤ 6	NA	NA	327	58%	327	58%
	> 6	NA	NA	237	42%	237	42%

For the Freiburg cohort, the median follow-up time was 8.7 years (range 5.5–14 years). Patients were predominantly male (66%). The median age at diagnosis was 62 years (interquartile range (iqr) 53–69 years). At diagnosis, 37% and 43% of patients could be assigned to the early, asymptomatic stages Rai 0 or Binet A, respectively. *IGHV* gene mutation status was available for 289 of the 316 patients. Of these, 114 (36%) had a non-mutated *IGHV* locus with ≥ 98% sequence homology, and 175 (55%) had a mutated *IGHV* locus. Cytogenetic analyses were available for 304 out of the 316 patients. In 138 cases (45%), a sole del(13)(q14) was detected; 38 patients (13%) had a del(11)(q22), 47 patients (15%) a trisomy 12, and 21 patients (7%) a del(17)(p13). Serum β2-microglobulin (B2MG) levels were available for 276 of the 316 patients. The median B2MG level was 3.29 mg/l (IQR 2.37–5.08 mg/l). One hundred and twenty-nine patients (41%) had an elevated B2MG level (upper limit of the normal range, 3.5 mg). According to CLL-IPI, 94 patients (30%) were classified as high (score = 2) or very high (score = 3) risk. Until the end of follow-up, 212 (67%) patients had received therapy.

Patients who were registered at the Augsburg University Hospital had a median follow-up time of 4.4 years (1.8–8.4 years). Patients were predominantly male (64%) and median age was 69 years (iqr 61–76 years). More than one-third of the cohort were in an early-stage Binet A or Rai 0 at diagnosis (35% and 63%, respectively). *IGHV* mutation status, cytogenetics, B2MG levels, and consequently the CLL-IPI score were not available for the majority of patients. Comorbidities were systematically recorded in the Augsburg cohort (summary in Supp. Figure 1), and patients were stratified using the Cumulative Illness Rating Scale (CIRS). A relevant comorbidity burden was identified in 327 (58%) patients with a CIRS > 6. Until the end of follow-up, 272 (48%) patients had received therapy.

### Overall survival

**Fig. 1:**
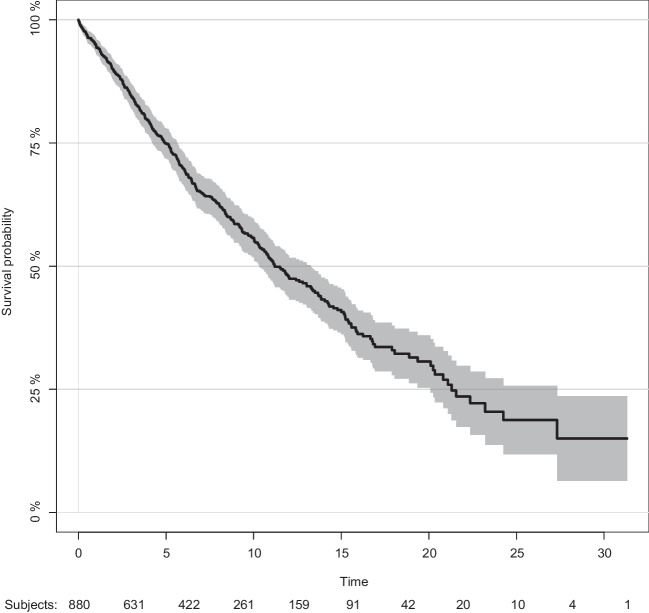
Overall survival (OS) of the entire cohort of CLL patients (*n* = 880) with a median follow-up of 7.3 years (iqr 2.7–10.1 years)

OS analysis included the entire cohort (*n* = 880) of CLL patients with a median follow-up of 6.0 years (iqr 2.7–10.1 years). Median OS was 11.2 years (CI 10.33–13.26 years; Fig. 1). The 5-year survival rate was 75% (CI 71.9–78%). Median OS was significantly different with 14.3 and 9.7 years (*p* = 1.68e-06) in the Freiburg and Augsburg cohort, respectively. As expected, OS of younger patients ≤ 65 years at diagnosis (*n* = 412) was significantly longer than in older patients (*n* = 468) with a 5-year survival rate of 87.9% (CI 84.5–91.2%) and 62.8% (CI 58–67.6%) and a median OS 16.8 years vs. 6.7 years [HR 3.00 (CI 2.41–3.72); *p* < 2e-16]. In contrast, no relevant differences for 5-year survival probability were observed for sex (Supp. Figure 2A, B). Stratification of OS by clinical stages showed the expected separation between early and advanced clinical stages (Supp. Figure 2C, D). Patients with Rai 0 vs. Rai I–IV had a 5-year survival probability of 87% (CI 83–91%) and 70.9% (CI 66.4–75.4%) years, respectively. Patients with stage Binet A had a superior median survival of 13.3 years compared to 6.7 years for Binet B/C patients. Elevated B2MG serum levels were indicative for dismal outcome with a 5-year survival probability of 90.9% (CI 86.1–95.6%) compared to 77.4% (CI 70.1–84.6%) for patients with normal range B2MG (Supp. Figure 2E).

**Fig. 2:**
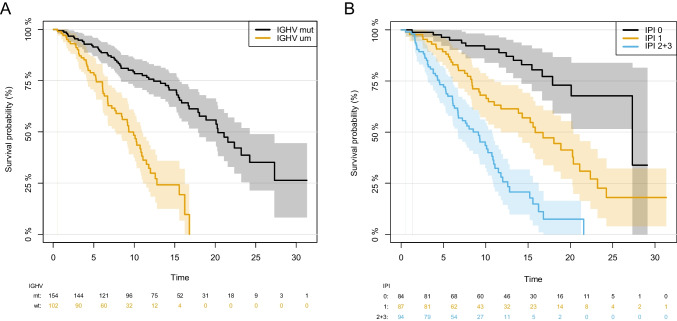
**A** Overall survival (OS) in the cohort of CLL patients separated by mutation status of the *IGHV* gene locus (mut, mutated; um, unmutated). **B** OS in the cohort separated into three risk groups (0, 1, and 2 + 3) as defined by the CLL-IPI score

For *IGHV* mutation status, we observed significant differences of 5-year survival probability with 91.4% (CI 87–95.9%) for mutated patients and 78.9% (CI 70.8–86.9%) for those with unmutated *IGHV* locus (Fig. 2A). Median OS was 20.3 years (CI 17.89–24.3) and 9.3 years (CI 7.77–10.9) for mutated and unmutated patients respectively [HR 3.90 (CI 2.58–5.88); *p* = 9.8e-11]. Patients with del(17)(p13) formed the patient subgroup with the poorest survival with a 5-year survival probability of 61.1% (CI 38.6–83.6%) compared to 87.6% (CI 83.5–91.8%) in patients with intact *TP53* (Supp. Figure 2F). We were also able to validate the prognostic separation of the composite risk score CLL-IPI in our non-study cohort of CLL patients (Fig. 2B). Patients with CLL-IPI scores 0 and 1 had a significantly better outcome with a median OS of 27.3 and 15.85 years (HR_1vs0_ 3.15, CI 1.77–5.60, *p* = 9.8e-5), respectively, compared to patients of the combined risk group 2/3 who had a median OS of 9.14 years (HR_2+3vs0_ 8.52, CI 4.84–15.01, *p* = 1.2e-13). Comorbidities as assessed by CIRS impacted significantly on prognosis. Patients with CIRS ≤ 6 had a significantly longer median OS compared to patients with higher comorbidity burden (HR_≤6vs>6_ 1.40, CI 1.07–1.84, *p* = 0.013) (Supp. Figure 2G).

### Conditional survival

**Fig. 3:**
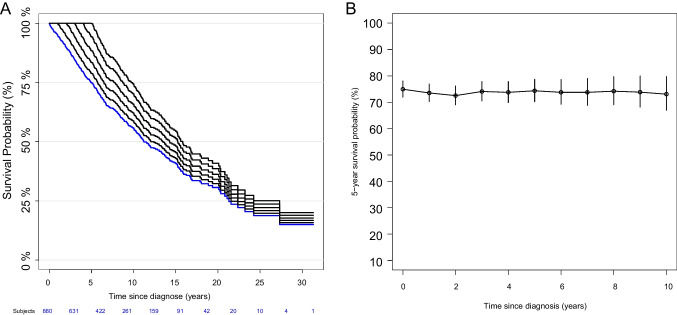
**A** Kaplan–Meier curves of conditional survival CS(*t*|*s*) for patients who have already survived *s* = 0 (blue), *s* = 1, to *s* = 5 years. **B** 5-year CS (CS(5|*s*)) rate depending on the *s* years already survived including 95% confidence intervals

CS was first determined for the entire patient cohort (*n* = 880) (Fig. 3A, B). The Kaplan–Meier curves for the landmarks *s* = 0 to *s* = 5 years showed parallel shifted comparable shapes, indicating highly similar survival of CLL patients in general, regardless of how long they had survived from diagnosis. The conditional 5-year survival CS(5|*s*) for *s* = 0 to *s* = 10 years after diagnosis ranged from 72.5% (CI 69–76.1%) to 74.9% (CI 71.9–78%) and remained constant throughout this period, indicating a stable prognostic prediction from baseline to 10 years after diagnosis. As a sensitivity analysis, we estimated CS incorporating center as a co-variable. Observed differences in CS were explained by the different age structures of the two community-based cohorts (Supp. Figure 3.A, B).

### Conditional survival stratified by prognostic factors

When separated by patient age (≤ 65 and > 65 years), a constant 5-year CS probability in the ranges of 79.4–85.7% and 60.2–66.0% was observed for both patient subgroups over the time span of *s* = 0,…,10 years after diagnosis. Patients ≤ 65 years at diagnosis (*n* = 412) had a higher probability for 5-year CS than older patients (Supp. Figure 4A). Throughout the observation period, we observed no sex-associated differences in CS (Supp. Figure 4B).

**Fig. 4:**
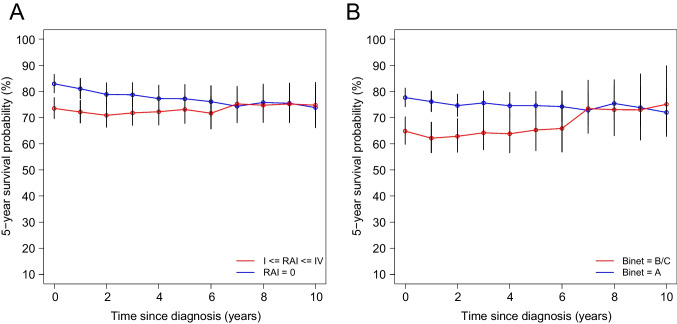
**A** Conditional survival (CS) depending on the clinical stage according to Rai classification (Rai 0, *n* = 314; Rai I–IV, *n* = 449). **B** CS according to Binet staging (Binet A, *n* = 494; Binet B + C, *n* = 289)

Patients in the early, asymptomatic stage Rai 0 (*n* = 314) at diagnosis had a significantly higher probability of 82.9%/81.0% vs. 73.5%/72.1% than higher stage patients for 5-year CS for the first 2 years (*s* = 0 and *s* = 1 years) after diagnosis (Fig. 4A). However, the 5-year CS probability of both subgroups converged in further years of the disease course and was almost identical between 73.8 and 75.8% between *s* = 7 and *s* = 10 years, indicating a loss of long-term prognostic value of assessment of Rai staging at diagnosis over time. An almost identical result was obtained by comparing the early, asymptomatic stage Binet A at diagnosis with the advanced disease stage Binet B/C (Fig. 4B).

**Fig. 5:**
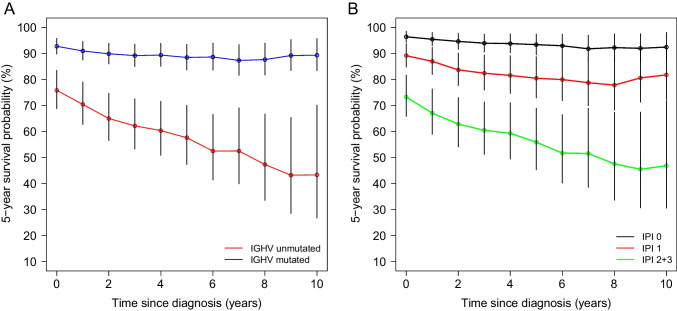
**A** Conditional survival (CS) of CLL patients separated by *IGHV* mutation (mutated, *n* = 208; unmutated, *n* = 170). **B** CS according to CLL-IPI risk groups (score 0/1/2 + 3: *n* = 314/449/117)

The strong prognostic value of *IGHV* mutation status was also reflected in the CS. Patients with a mutated *IGHV* locus had a stable 5-year CS between 87.3 and 92.8% without significant changes from baseline over the time span of 10 years from diagnosis (*s* = 0 to *s* = 10 years). In contrast, high-risk patients with unmutated *IGHV* locus showed a significantly lower 5-year survival probability of 75.8% at diagnosis (*s* = 0 years) with an immediate steady decline to 43.3% over the identical time span (Fig. 5A). This was also evident in the analysis of CLL-IPI (Fig. 5B). While patients with CLL-IPI 0 had a stable 5-year CS of 96.4, 93.4, and 92.5% over the landmarks from *s* = 0, *s* = 5, to *s* = 10 years, a trend of a decline from baseline at diagnosis could be observed for CLL-IPI 1 patients with 89.2, 80.5, and 81.8% over the identical landmarks. In high-risk patients with CLL-IPI 2/3, 5-year CS was already significantly lower at 73.3% at the time of diagnosis and showed a significant decline to 46.9% over the entire 10-year period after diagnosis. A similar, yet less pronounced, decrease in 5-year CS over the course of 10 years after diagnosis was also observed for high-risk patients defined by del(17)(p13) (Supp. Figure 3C). The extent of comorbidities proved to be a prognostic factor in the Augsburg cohort with a 5-year CS at the time of diagnosis of 73.6% and 65.1% for patients with CIRS ≤ 6 and CIRS > 6, respectively. However, the 5-year CS during the observation period showed almost perfectly parallel curves for both subgroups with no significant changes over the 10-year period (see Fig. 3D).

Finally, we asked if receiving treatment over the observation period would impact CS. Patients who had received treatment at any time point during the observation period had a worse prognosis at diagnosis than untreated patients, with 56.8 vs. 76.2% 5-year survival. However, receiving treatment for CLL was not associated with significant changes in CS over time (Supp. Figure 4).

## Discussion

Accurate long-term prognosis in CLL is a challenge. Existing prognostic models, such as the CLL-IPI, are limited as they provide predictions based on a single time point, typically at diagnosis, without taking into account years already survived. This limitation hinders effective disease management and appropriate counseling of CLL patients. Previous analyses of prognostic models in CLL have highlighted this issue, raising uncertainties about which models can be reliably used in clinical practice to predict long-term outcomes [15].

In this study, we present a systematic analysis of absolute CS in CLL patients based on data from a non-study cohort comprising individuals diagnosed between 1984 and 2021 at two university medical centers. Our goal was to investigate survival estimates over time, stratified across different CLL patient subgroups. With a mean post-diagnostic follow-up of 7.3 years, we observed a constant 5-year CS of approximately 75% for the entire patient cohort, demonstrating stable survival over a period of up to 10 years.

Notably, CS remained remarkably stable regardless of age at diagnosis, although patients older than 65 years exhibited approximately 20% lower CS likelihood. These findings are consistent with a Canadian study that demonstrated stable CS up to 5 years after diagnosis [19]. Data from the USA and the Netherlands noted very slight decreases over time [20, 21]. However, it should be noted that the analysis in these studies examined relative CS from an epidemiological perspective, whereas we emphasized the patient-relevant perspective and considered absolute CS. These data suggest that the probability of surviving additional 5 years remains at 75% or slightly below over the disease course, indicating that CLL patients face a constant risk of death with each additional year of survival [22]. This is in contrast to the patterns observed in most hematologic and solid malignancies that are potentially curable [23]. Some aggressive diseases (e.g., pancreatic cancer, malignant melanoma) are associated with increasing CS [14, 17]. For other entities at early stages or with a tendency to recur (e.g., prostate or breast cancer), CS increases slightly over time or remains stable [19, 20, 23]. Constant CS comparable to CLL has been shown for multiple myeloma [24]. Common to both entities is the lack of curative treatment options and the goal of remission maintenance. CLL remains incurable to date with a steady risk of infection, autoimmune complications, secondary malignancies, and conversion to high-grade B-cell lymphoma (Richter’s transformation). It is unclear whether the availability of highly effective targeted treatment options (e.g., BTKis, venetoclax, novel CD20 antibodies) might influence these results.

Previous analyses have mostly not included the clinical and biological heterogeneity of CLL. Because such factors are available for a large proportion of patients in our cohort, we were able to stratify patients by known risk parameters including the composite prognostic index CLL-IPI. For *IGHV* mutation status, *TP53* deficiency, and the CLL-IPI, our CS analyses revealed a clinically meaningful and significant separation of subgroups. *IGHV* mutation status and *TP53* deficiency have known prognostic value and impact on the choice of targeted therapy [25, 26]. Their importance is also emphasized by the weighting in the CLL-IPI scoring system. The much less favorable prognosis of patients with an unmutated *IGHV* locus and the marked deterioration over time (CS decreases by approximately 30% over 10 years) not seen in *IGHV*-mutated patients reflect the heterogeneity of CLL and the fundamental biological differences of the disease associated with the *IGHV* mutation status. While patients with del(17)(p13) had a similarly poor prognosis at diagnosis followed by worsening in CS over time, the small number of patients and possible acquisition of *TP53* deficiency during disease progression could obscur prognostic trends over time.

Not surprisingly, the composite prognostic score CLL-IPI, whose “static prognostic significance” we can excellently reproduce here in a “real-world” cohort outside clinical trials, shows a similar prognostic separation of CS over time with a 5-year survival rate ranging from a stable 95% (CLL-IPI = 0) to a decline to 25% (CLL-IPI = 2 + 3) over the 10-year observation period.

Patients in need for treatment over the observation period were less likely to survive, whereas 5-year CS increased slightly over time for untreated patients. This finding aligns with a recent study from the USA, which reported an increase in 5-year CS for untreated CLL patients aged ≥ 66 years based on linked surveillance, epidemiology, and end results-Medicare data [27]. However, given the heterogeneity of the patient cohort, the length of the observation period, and the therapeutic advances that have led to substantial changes in treatment regimens over time, meaningful conclusions are difficult.

In patients with CLL and other cancers, comorbidity is associated with shorter survival [28–31]. In CLL, comorbidity has been shown to be an independent predictor of outcome, and different types of comorbidities are associated with increased overall mortality and particularly higher CLL-related mortality [32]. While the extent of comorbidities also had a significant impact on prognosis in our cohort, as previously reported for CLL, CS probability did not show significant changes over the 10-year period when stratified by CIRS score, using a cutoff commonly used in clinical trials to identify patients with relevant comorbidity burden (≤ 6 vs. > 6). This indicates that the prognostic significance of comorbidities remains similarly relevant and does not decline with increasing disease duration. This might be connected to a significant interaction between comorbidities and CLL treatment (in terms of treatment options and treatment tolerance) as previously demonstrated [29].

Thus, in order to profoundly investigate the influence of comorbidities on prognosis and CS in CLL patients in more detail, it is relevant to identify the causes of mortality (CLL related vs. unrelated), as CLL-related deaths also contribute significantly to increased mortality in patients with a high burden of comorbidities. This is mainly due to the fact that increased comorbidities are associated with a reduced chance of sufficient disease control [29]. However, the documentation of causes of death was only very incomplete in our registry-based dataset and therefore does not allow any analyses in this regard. This clearly demonstrates that inclusion and documentation of comorbidities and, in particular, causes of death in cancer registries, are essential for a meaningful prognosis assessment at time of diagnosis and for dynamic CS assessment over the disease course.

The study has several other limitations. First, the limited number of patients restricts detailed subgroup analyses and statistical power. Additionally, molecular parameters were only available for a subset of patients, potentially limiting the scope of the findings. Second, no reassessment of prognostic parameters, such as clinical stage or genetic changes, was performed. The extended 35-year period of diagnosis and follow-up, with data collected independently in two cohorts, may introduce variability in the analyses. In addition, patient and disease characteristics of patients from the two university hospitals comprising the cohort may not be fully comparable, and there is a risk of a bias towards patients with higher complication rates and more comorbidities.

In addition, at later time points in the patient observation period, there were newer treatments available that were not approved at the beginning. Thus, the effects of treatment remain largely unclear because of the fundamentally different types of therapies administered. Stratification by type of therapy or by time windows encompassing different modes of disease management (e.g., pre-Rituximab era or post 2014 introduction of BTKis) was not possible because of the cohort size and limited follow-up. As targeted therapies have become increasingly important in treatment regimens in recent years, their impact on current prognostic models remains to be determined. Finally, no information is available on the specific causes of death, which precludes conclusions about the reasons for persistent excess mortality in the entire cohort and increasing mortality in high-risk patients.

### Summary and conclusion

Overall, we can demonstrate that CS is relevant for the management of CLL and the assessment of prognosis for physicians and patients. We confirm the previously reported stable prognosis of CLL patients over a long observation period and show that high-risk subgroups undergo dramatic and patient-relevant prognostic changes over time with gradually increasing mortality. In a disease like CLL, which often progresses slowly over decades, this type of prognostic information is clearly superior to a static survival model and of higher relevance to the patient.

### Supplementary Information

Below is the link to the electronic supplementary material.

Supplementary file1 (PDF 333 KB)

Supplementary file2 (PDF 25 KB)

## Data Availability

Data available on request from the authors.
