# Supplementary material for: Conditional survival to assess prognosis in patients with chronic lymphocytic leukemia
Source: Ann Hematol. 2024 Feb 3;103(5):1613–22. doi: 10.1007/s00277-024-05627-w (PMC11009732; doi:10.1007/s00277-024-05627-w)
Supplement: Supplementary file 1 — Supplementary file1 (PDF 333 KB) [file 277_2024_5627_MOESM1_ESM.pdf]

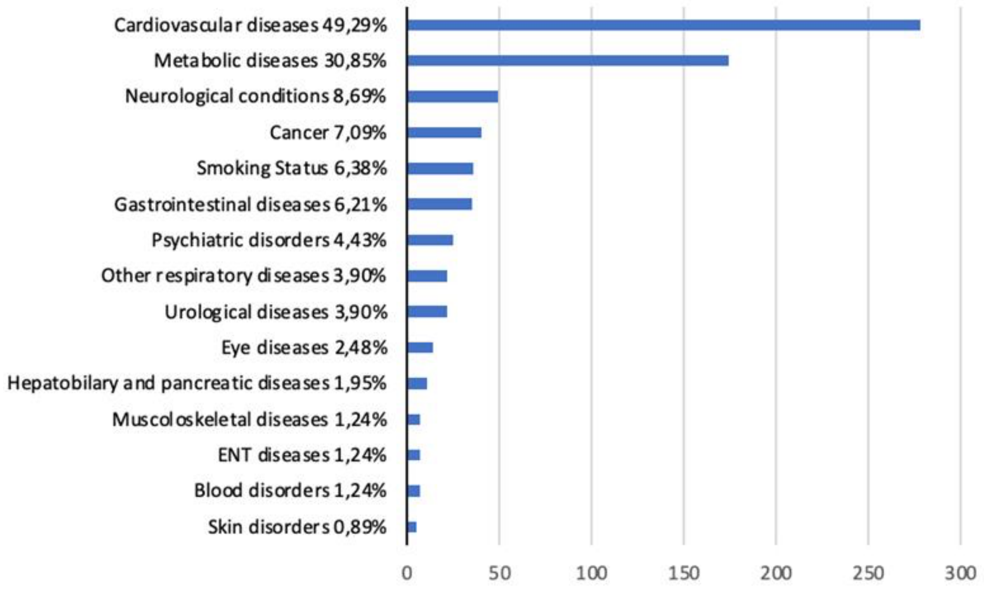

Supp. Figure 1: Frequency of comorbidities/comorbidity groups for patients of the Augsburg subcohort (n=564).

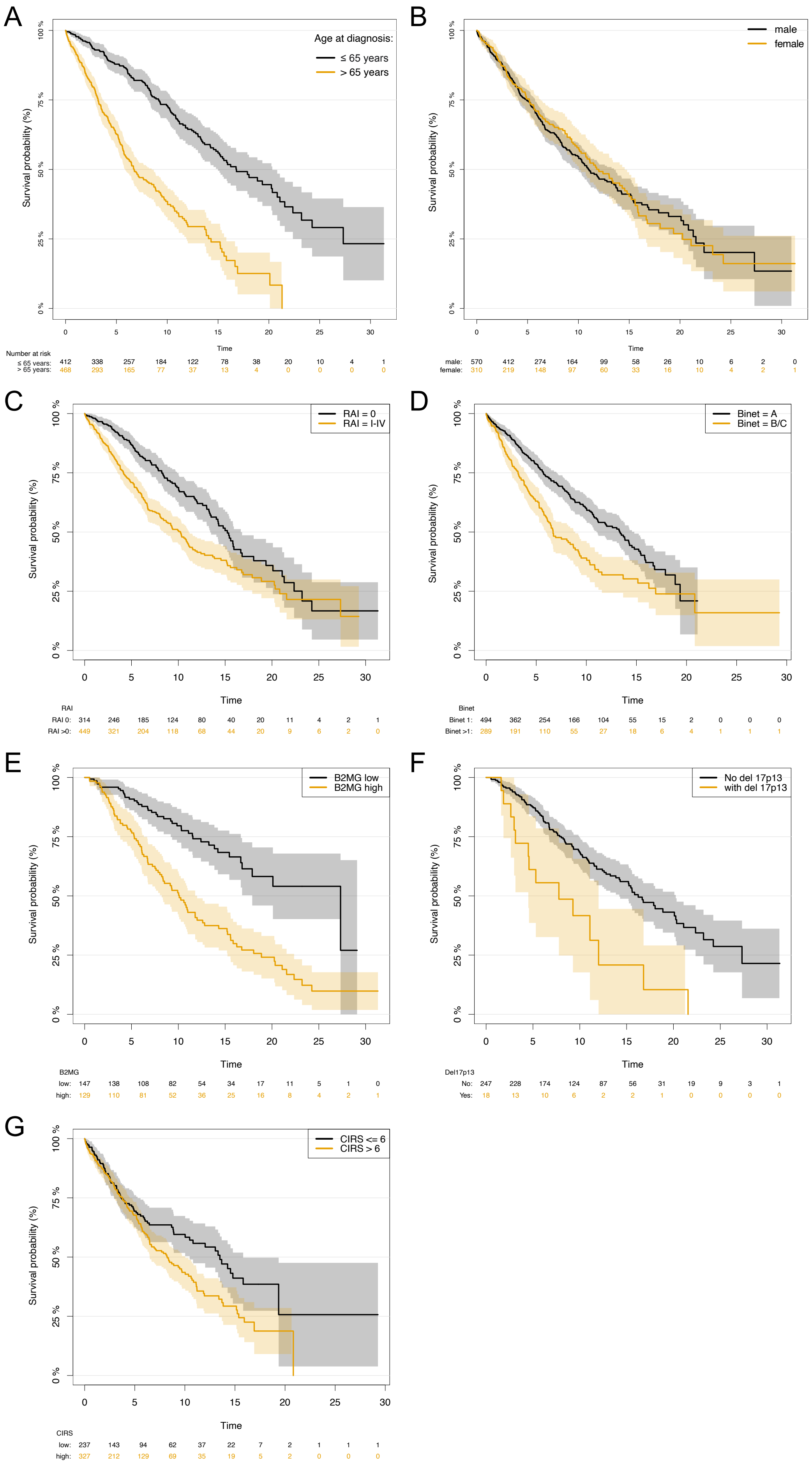

A
Age at diagnosis:
≤ 65 years
> 65 years
Survival probability (%)
Time
Number at risk
≤ 65 years: 412 338 257 184 122 78 38 20 10 4 1
> 65 years: 468 293 165 77 37 13 4 0 0 0 0
B
male
female
Survival probability (%)
Time
male: 570 412 274 164 99 58 26 10 6 2 0
female: 310 219 148 97 60 33 16 10 4 2 1
C
RAI = 0
RAI = I-IV
Survival probability (%)
Time
RAI
RAI 0: 314 246 185 124 80 40 20 11 4 2 1
RAI >0: 449 321 204 118 68 44 20 9 6 2 0
D
Binet = A
Binet = B/C
Survival probability (%)
Time
Binet
Binet 1: 494 362 254 166 104 55 15 2 0 0 0
Binet >1: 289 191 110 55 27 18 6 4 1 1 1
E
B2MG low
B2MG high
Survival probability (%)
Time
B2MG
low: 147 138 108 82 54 34 17 11 5 1 0
high: 129 110 81 52 36 25 16 8 4 2 1
F
No del 17p13
with del 17p13
Survival probability (%)
Time
Del17p13
No: 247 228 174 124 87 56 31 19 9 3 1
Yes: 18 13 10 6 2 2 1 0 0 0 0
G
CIRS <= 6
CIRS > 6
Survival probability (%)
Time
CIRS
low: 237 143 94 62 37 22 7 2 1 1 1
high: 327 212 129 69 35 19 5 2 0 0 0

Supp. Figure 2: (A) OS stratified by age (>65 years) with a median OS 16.8 years vs. 6.7 years [HR 3.00 (CI 2.41-3.72); p=4.4e-23]. **(B)** OS stratified by sex with a 5-year survival rate of 75.4% (CI 70.2%-80.5%) and 74. 7% (CI 70.9%-78.6%) [HR 0.97 (CI 0.79-1.20); p=0.811]. **(C)** Stratification of OS by clinical Rai stages. Patients with Rai 0 vs. Rai I-IV had a 5-year survival probability of 87% (CI 83%-91%) and 70.9% (CI 66.4% 75.4%) years [HR 1.64 (CI 1.31-2.07); p=2.0e-5]. **(D)** Patients with stage Binet A had a superior median survival with 13.3 years compared to 6.7 years for Binet B/C patients [HR 1.71 (CI 1.38-2.13); p=1.3e-6]. **(E)** Elevated B2MG serum levels are highly indicative for dismal outcome with a 5-year survival probability of 90.9% (CI 86.1%-95.6%) compared to 77.4% (CI 70.1%-84.6%) for patients with normal range B2MG [HR 2.86 (CI 1.97-4.14); p=2.9e-8]. **(F)** Patients with del(17)(p13) formed the patient subgroup with the poorest survival within our cohort with a 5-year survival probability of 61.1% (CI 38.6%-83.6%) compared to 87.6% (CI 83.5%-91.8%) in patients with intact *TP53* [HR 1.90 (CI 1.16-3.11); p=0.011]. **(G)** 5-year survival probabilities according to CIRS score [HR 1.40 (CI 1.07-1.84); p=0.013].

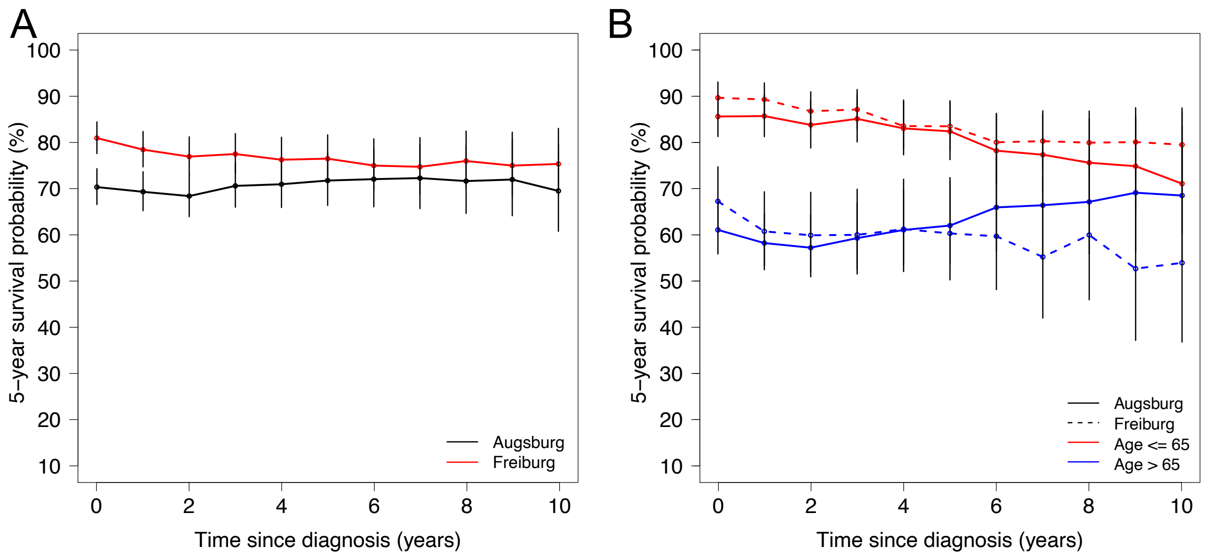

Supp. Figure 3: (A) 5-year conditional survival of the entire CLL cohort (n=880) patients incorporating medical center (Augsburg versus Freiburg) as a co-variable **(B)** 5-year conditional survival of the entire CLL cohort (n=880) patients adding age (≤65 years versus >65 years at diagnosis) as a further co-variable.

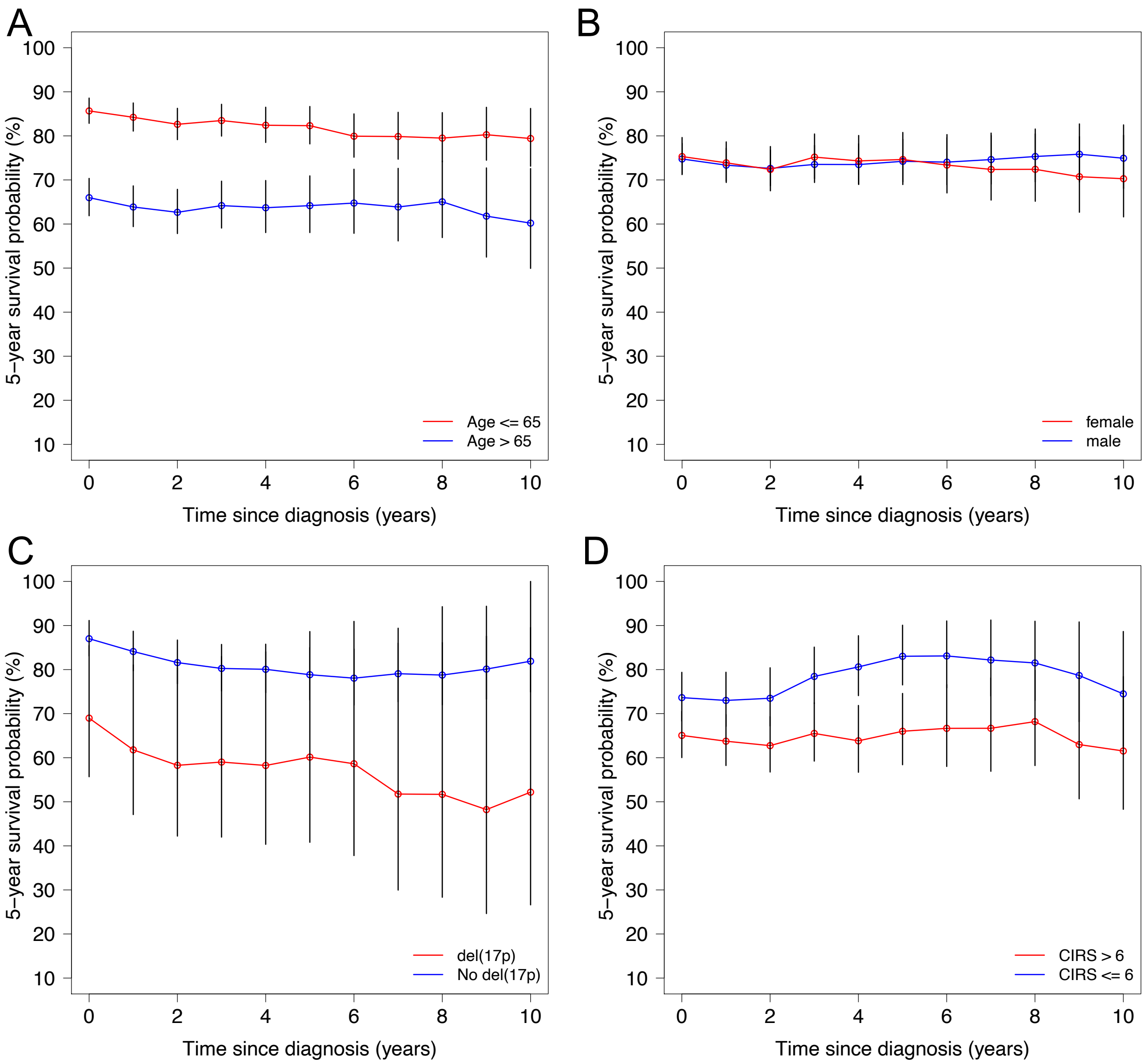

Supp. Figure 4: (A) Conditional survival of the entire CLL cohort (n=880), stratified by age (≤65 years versus >65 years at diagnosis), given as the 5 year-survival rate depending on the s years already survived. **(B)** 5-year conditional survival of the entire CLL cohort (n=880) stratified by sex. **(C)** 5-year conditional survival over the course of 10 years after diagnosis stratified by cytogenetic risk (presence versus absence of del(17)(p13)) for n=357 CLL patients with available cytogenetics. (**D**) 5-year conditional survival stratified by extent of comorbidities (CIRS ≤6 versus >6 at diagnosis) for n=564 CLL patients with available information on comorbidities.

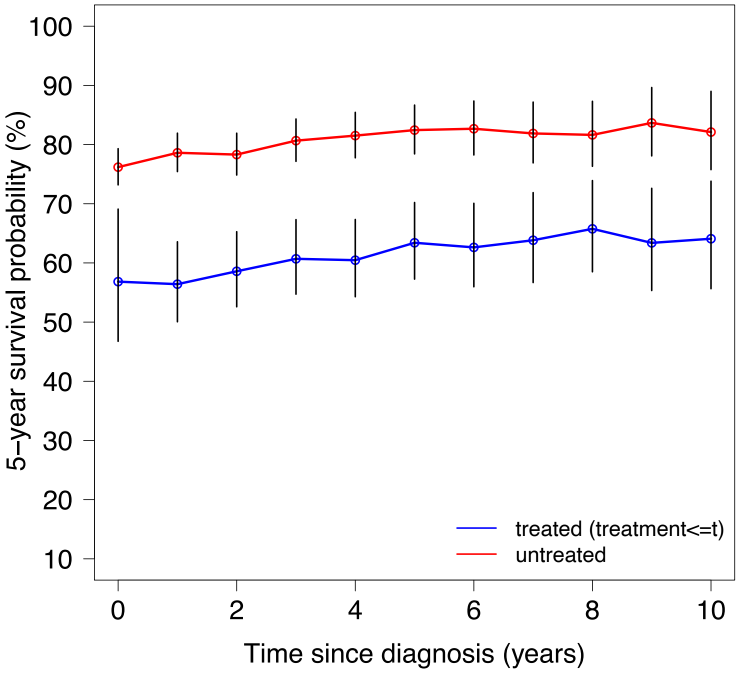

Supp. Figure 5: 5-year CS probability over the landmarks s=0 to s=10 years from baseline for patients having received treatment (treated) versus patients not having received any treatment over the observation period (untreated).
