# Supplementary material for: Conditional survival to assess prognosis in patients with chronic lymphocytic leukemia
Source: Ann Hematol. 2024 Feb 3;103(5):1613–22. doi: 10.1007/s00277-024-05627-w (PMC11009732; doi:10.1007/s00277-024-05627-w)
Supplement: Supplementary file 2 — Supplementary file2 (PDF 25 KB) [file 277_2024_5627_MOESM2_ESM.pdf]

Supplemental Methods

Assessment of biological and molecular biomarkers

Beta-2-microglublin (B2MG) was measured in serum as part of routine clinical practice. Quantification (mg/l) was performed according to standardized laboratory tests, normal range for B2MG was defined as ≤3.5 mg/l.

Genomic aberrations were detected by fluorescent in situ hybridization (FISH) according to local standard procedures. At least 200 interphase cells were examined. Deletion (del) 17p was used as the sole marker of *TP53* deficiency.

IGHV mutation status was assessed according to local standards based on the procedure described previously. *IGHV* sequence homology of less than 98% compared to germline was considered mutated.
